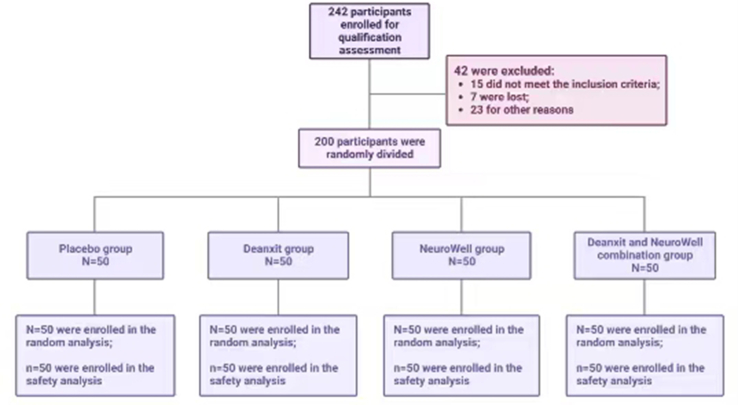# The efficacy and safety of NeuroWell antidepressant dietary supplement, Deanxit, and their combination in the treatment of mild-to-moderate depression: A randomized clinical trial

**DOI:** 10.1016/j.gendis.2023.101171

**Published:** 2023-12-13

**Authors:** Honglei Yin, Zhuxin Sui, Yutong Wang, Dechen Liu, Qinglu Wang, Yanchao Wang, Lei Sun, Jinfeng Li, Zhen Li, Yue Liu, Shang Guo, Wenwen Wang, Hongtao Yin, Ping Liu, Kezhong Zhang, Huaibo Zhang, Yajun Liu, Yanqiu Liu, Qiqi Zhou, Dianfang Wei, Hui Zhang, Shanshan Wang, Yaochao Ning, Shuang Geng, Xuejun Wen, Youping Deng, Hongwei Wang, Yunliang Wang

**Affiliations:** a Neurology Department, ZiBo 148 Hospital, China RongTong Medical Healthcare Group Co., Ltd., Zibo, Shandong 255300, China; b Department of Histology and Embryology, Teaching Department of Basic Medicine, Qilu Medical University, Zibo, Shandong 255300, China; c School of Public Health and Laboratory, Qilu Medical University, Zibo, Shandong 255300, China; d School of Medicine, The Henan University of Chinese Medicine, Zhengzhou, Henan 450046, China; e College of Sport and Health, Shandong Sport University, Jinan, Shandong 250102, China; f Institute of Oncology, The Fifth Medical Centre, Chinese PLA General Hospital, Beijing 100071, China; g School of Nursing, Qilu Medical University, Zibo, Shandong 255300, China; h Department of Orthopedic Surgery, Shanghai Sixth People's Hospital Affiliated to Shanghai Jiao Tong University School of Medicine, Shanghai 200023, China; i Office of Infection Prevention and Control, The Fifth Clinical Medical College of Henan University of Chinese Medicine (Zhengzhou People's Hospital), Zhengzhou, Henan 450002, China; j Department of Neurology, Zibo Central Hospital, Zibo, Shandong 255036, China; k Department of Neurology, The First Affiliated Hospital of Nanjing Medical University, Nanjing, Jiangsu 210029, China; m Research Assistant Professor Center for Alcohol Research in Epigenetics, Department of Psychiatry, University of Illinois at Chicago, Chicago, IL 60612, USA; n Department of Pharmacology, School of Pharmacy, Qilu Medical University, Zibo, Shandong 255300, China; o Department of Rehabilitation Medicine, Zibo First Hospital, Zibo, Shandong 255200, China; p XREGEN Research Institute, Hangzhou, Zhejiang 310056, China; q School of Life Sciences, Zhengzhou University, Zhengzhou, Henan 450001, China; r Key Laboratory of Spine and Spinal Cord Injury Repair and Regeneration of Ministry of Education, Orthopedic Department of Tongji Hospital, School of Life Science and Technology, Tongji University, Shanghai 200065, China; s International Institute for Biomedical Biomaterials (I2BM), Zhengzhou, Henan 450018, China; t Department of Chemical and Life Science Engineering, School of Engineering, Virginia Commonwealth University, Richmond, VA 23284, USA; u Department of Quantitative Health Sciences, John A. Burns School of Medicine, University of Hawaii at Manoa, Honolulu, HI 96813, USA; v Department of Medicine, The University of Chicago, Chicago, IL 60637, USA; w Neurology Department, The Second Affiliated Hospital of Zhengzhou University, Zhengzhou, Henan 450014, China

Depression is the leading global cause of disability, affecting about 300 million people worldwide.^1^^,^^2^ Depending on the number and severity of symptoms, depressive episodes can be classified as mild, moderate, and severe. Previous studies have typically focused on the treatment of severe refractory depression, while there have been few studies on the treatment of mild-to-moderate depression. However, patients with mild-to-moderate depression may develop severe episodes if not properly treated. Antidepressant dietary supplements have been suggested as an alternative treatment for depression, but the effectiveness of these supplements varies widely, and their efficacy has not been rigorously tested in clinical trials. NeuroWell contains all plant extracted ingredients and affects multiple pathways to provide a multi-targeted treatment. Preliminary results indicated that NeuroWell is safe and meets the U.S. FDA standards for dietary supplements. NeuroWell is currently in the U.S. market to relieve depression and anxiety and has exhibited remarkable clinical effects. However, no systematic clinical trials have been performed to investigate the effects of NeuroWell on mild-to-moderate depression and anxiety. Deanxit is an antidepressant widely used in the treatment of depression. It is a mixture of flupentixol and melitracine, which increases the concentration of neurotransmitters in the intracerebral synaptic space. It is mainly used in patients with mild-to-moderate anxiety and depression with major somatization symptoms.^3^, ^4^, ^5^ Thus, Deanxit was selected as the positive control for NeuroWell in this clinical trial.

To test the safety of the NeuroWell dietary supplement, we first conducted acute toxicity and subacute toxicity tests in animals. There were no changes in the body weight of mice in the acute toxicity test. During the subacute toxicity tests, rats in each group were in good mental states and no death occurred. There were no significant differences in blood routine indexes (Table S1). Furthermore, a series of behavioral tests were carried out to clarify the intervention effect of NeuroWell on depression compared with Deanxit. Compared with the normal control group, the 14-day body weight gain of each administration group was significantly higher than that of the control model group (Fig. 1A). The sugar water consumption (Fig. 1B), the total distance traveled (Fig. 1C), and the tail suspension time (Fig. 1D) of NeuroWell-treated rats in chronic unpredictable mild stress rat model of depression were all significantly improved. Based on these, we carried out clinical trials to further clarify the therapeutic effects of the NeuroWell dietary supplement on depression.

**Figure 1:**
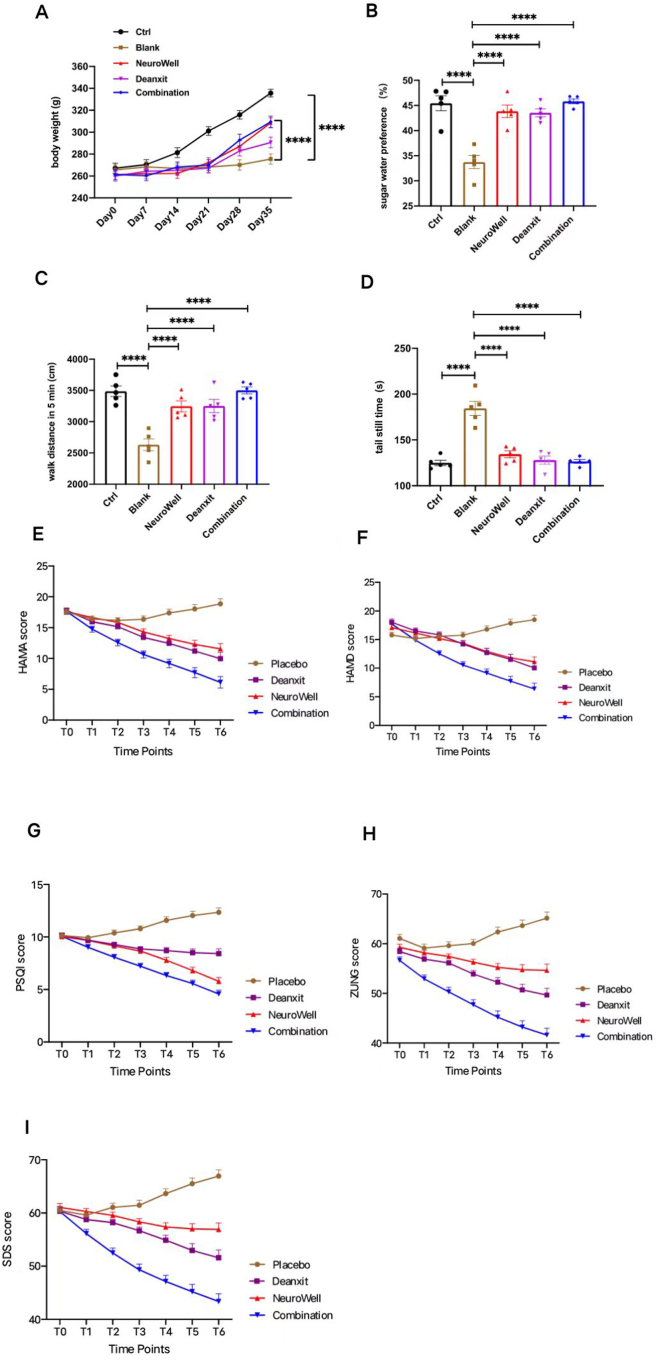
Safety and Effective analysis of Deanxit, NeuroWell, and their combination groups by various parameters. **(A**–**D)** Behavioral tests of the rats include (A) body weight measurement, (B) sugar water consumption analysis, (C) walk distance in 5 min, and (D) time measurement of rat tail holding time. **(E**–**I)** Score analyses of the four groups of participants enrolled include (E) Hamilton anxiety scale (HAMA) score analysis, (F) Hamilton depression scale (HAMD) score analysis, (G) Pittsburgh sleep quality index (PSQI) score analysis, (H) Zung's self-rating anxiety scale score analysis, and (I) Zung's self-rating depression scale score analysis.

Then, a total of 242 participants were recruited, and 200 were eventually enrolled and randomly grouped (*n* = 50) for the study (Fig. S1). None of the patients in any of the four groups had dropped out by 12 weeks. The general data for the four groups were comparable, with no statistically significant difference (Table S2). The impact of the interventions on the outcome measures was assessed at seven time points. The duration of treatment had a significant influence on the outcomes (F = 41.844–97.487, all *P* < 0.001). In addition, significant interactions were noted for all outcome measures, as was expected (F = 21.283–41.329, all *P* < 0.001).

As shown in Figure 1E and F, the results of the four groups changed over time, the Deanxit, NeuroWell, and the combination groups showed a linear downward trend for the seven time points (*P* < 0.05). Both the Hamilton anxiety scale and Hamilton depression scale scores were reduced (*vs*. baseline/before treatment) in the Deanxit and NeuroWell groups compared with the placebo. The largest decreases were seen in the combination (Deanxit + NeuroWell) group, while the NeuroWell and Deanxit groups showed similar improvements for both the Hamilton anxiety scale and Hamilton depression scale scores.

Figure 1G shows that the Pittsburgh sleep quality index was reduced in both the Deanxit and NeuroWell alone groups, while the strongest effects were seen in the combination group. The changes in the NeuroWell group and the Deanxit group overlapped from week 2 to week 6, indicating that the improvements in sleep were similar in the two groups. However, the decrease in the Pittsburgh sleep quality index was more significant for the NeuroWell group beginning from week 8, indicating that the effects of the NeuroWell on sleep disorders were better than those of Deanxit in the longer term.

Both Deanxit and NeuroWell reduced the Zung's self-rating anxiety scale score and self-rating depression scale score. Consistent with the other parameters, stronger effects were seen in the combination group. At the beginning of weeks 2, 4, and 6, the curves of the NeuroWell group and the Deanxit group were similar, indicating that NeuroWell was as effective as Deanxit in improving anxiety and depression. The changes recorded in weeks 8, 10, and 12 showed that the curve of the NeuroWell group was still slowly decreasing, although less than the Deanxit group. The combination group continues to show more effective improvement than either treatment alone up to week 12.

On day 1, 13 (26%) participants in the Deanxit group and 8 (16%) in the combination group reported dry mouth and constipation. On day 3, 14 participants (28%) in the Deanxit group and 10 (20%) in the combination group had dry mouth and constipation (28%). After 3–12 weeks of treatment, 18 participants (36%) in the Deanxit group and 11 (22%) in the combination group reported experiencing dry mouth and constipation. These adverse reactions were not reported in the NeuroWell or placebo groups (Table S2). No other adverse effects (headache, fatigue, nausea, or rash) were noted in any of the groups. In addition, the results of routine blood and urine tests (*e.g.*, alanine transaminase, aspartate aminotransferase, blood urea nitrogen, and creatinine) were within the normal ranges, indicating that there were no major adverse effects on the liver or kidneys.

These data indicate that NeuroWell is safe and reliable for treating depression, has a rapid onset, produces lasting effects, and has no apparent side effects. It can improve depression-associated sleep disorders and sleep quality, while also reducing anxiety and depression. Importantly, NeuroWell was found to be more effective in improving sleep disorders compared with Deanxit.

## Ethics declaration

The human study was approved by Medical Ethics Committee of Qilu Medical College. For age- and sex-matched rats, animals were fed and maintained under specific pathogen-free conditions following the criteria of the National Institutes of Health (Bethesda, MD) Guide for the Care and Use of Laboratory. Animal treatments received the approval of the ethics committees of Qilu Medical College.

## Author contributions

Y.L.W., X.J.W., and Y.P.D. conceived and designed the experiments; D.C.L. and X.Z.S. analyzed the data; Y.T.W. contributed reagents/materials/analysis tools; H.L.Y., H.W.W., and X.J.W. wrote the paper; Y.L.W., Y.T.W., X.J.W., and H.W.W. contributed to the interpretation of the results and the revision of the manuscript.

## Conflict of interests

The authors declare no competing interests.

## Figures and Tables

**figs1:**