# Supplementary material for: The efficacy and safety of NeuroWell antidepressant dietary supplement, Deanxit, and their combination in the treatment of mild-to-moderate depression: A randomized clinical trial
Source: Genes Dis. 2023 Dec 13;11(6):101171. doi: 10.1016/j.gendis.2023.101171 (PMC11298832; doi:10.1016/j.gendis.2023.101171)
Supplement: Multimedia component 1 [file mmc1.docx]

**Supplementary Materials**

**Methods**

**Animal experiments**

**Acute Toxicity Test**

60 mice were selected for the experiment, weighing ~20 g, half male and half female, and the females were not pregnant. They were divided into three groups: NeuroWell antidepressant group, Deanxit group, and blank control group. Before the test, each mouse was fasted for 12 hours without water. Mice in the NeuroWell group were orally given 30 mg once within 24 hours, and mice in the Deanxit group were given 6 mg once within 24 hours. The blank control group was given drinking water, observed continuously for 7 days, and fed routinely. Pay attention to animal activities, coat color, eating and defecation, and record the number of dead animals and the time of death. The dead animals were dissected, and if abnormalities were found with the naked eye, a pathological examination should be carried out.

The normal dose of NeuroWell is 1000 mg (1 g) per day for human adults (average body weight 65 kg), and 0.3 mg per day for mice with an average body weight of 0.02 kg (20 g). Mice were given 30 mg of NeuroWell a day, equivalent to 100 times the human dose. Observe the diet, defecation, mental state, animal death, and weight changes of animals in the NeuroWell, Deanxit and blank control groups within 1 week after administration of the test substances.

**Subacute Toxicity Test**

140 SD rats were randomly divided into 7 groups: blank control group, high, medium and low dose NeuroWell antidepressant groups (770, 390, 153 mg/kg per day, 20 rats in each group, half male and half female), low, medium and High-dose Deanxit groups (30mg, 60mg, 150mg/kg per day, 20 rats in each group, half male and half female) were fed antidepressant powder samples for 7 days, and the control group was given the same amount of starch powders. During the administration of test samples, the general clinical manifestations of animals in each group were observed every day, and the signs, degree and duration of poisoning and death of animals were recorded. Body weight and food intake were weighed once before sampling and before sacrifice. The animals were sacrificed 1 week after the samples were given, and fasted for 12 hours before the sacrifice. During the test samples, the general clinical manifestations of the animals in each group were observed every day, and the signs, degree, duration and death of the animals were recorded. The observation contents include coat, skin, eyes, mucous membranes, secretions, excretions, respiratory system, nervous system, autonomous activities and behavioral performance. Body weight and food intake were weighed once before sampling and before sacrifice. The animals were sacrificed 7 days after the samples were given, fasted for 12 hours before sacrifice, blood was collected for routine blood examination (hemoglobin, platelet count, red blood cell count, white blood cell count and classification), and then the animals were sacrificed and dissected. Weigh the heart, liver, spleen, lung, kidney, adrenal gland, thymus, testis, and ovary and calculate organ coefficients. Blood biochemical indicators: alanine aminotransferase (ALT), aspartate aminotransferase (AST), blood urea nitrogen (BUN), creatinine (Cr), blood glucose (Glu), serum albumin (Alb), total protein (TP), total cholesterol ( Determination of TCH), alkaline phosphatase (ALP), total bilirubin (TBIL), indirect bilirubin (D-BIL), triglyceride (TG), potassium, sodium, chloride, and calcium. The spinal cord, brain, optic nerve, pituitary gland, heart, liver, spleen, pancreas, lung, kidney, adrenal gland, thymus, lymph node, sternum, bladder, testis, epididymis, prostate, uterus, ovary, stomach, and intestine were collected for histopathological examination.

**Behaviors of Depression Mouse Model**

Through the establishment of rat chronic mild unpredictable stress (chronic unpredictable mild stress, CUMS) model, the rats were randomly divided into a normal control group, model control group (no drug administration), NeuroWell treatment group (15.3mg/100g), Deanxit treatment group (0.3 mg/100g), NeuroWell plus Deanxit treatment group (15.3mg/100g, 0.3mg/100g,), each administration group was given by intragastric administration for 14 days. Observe the differences in HE-stained hippocampal CA1 area of rats, open field tests (to detect the total distance traveled by rats in 5 minutes), sugar water preference tests (calculate the percentage of sugar water), emotional losses, and tail suspension tests.

**Preparation of the rat CUMS depression model**

Briefly, rats in the normal control group were supplied with normal feed and drinking water (except water deprivation for 24 hours before the 1% sucrose water consumption experiment) and did not receive any stimulation. The other groups received 35 days of chronic mild and unpredictable stress stimuli, mainly including ice water swimming, day and night reversal, binding and restraint for 3 hours, and tail clamping for 2 minutes, shaking, fasting for 24h and water deprivation for 24h. A total of 7 stimulation methods were given randomly every day, and the same stimulation method was used no more than 5 times in total for 35 days. The animals could not predict the stimulation they would encounter. On the 7th, 14th, 21st, and 35th day of the modeling process, the body weight of the experimental animals was weighed with an electronic balance.
