# Supplementary material for: The efficacy and safety of NeuroWell antidepressant dietary supplement, Deanxit, and their combination in the treatment of mild-to-moderate depression: A randomized clinical trial
Source: Genes Dis. 2023 Dec 13;11(6):101171. doi: 10.1016/j.gendis.2023.101171 (PMC11298832; doi:10.1016/j.gendis.2023.101171)

**Supplementary Materials**

**Materials and Methods**

**Participants**

Patients with mild or moderate depression treated in the 148th Hospital, Zibo, from January 2021 to August 2022, were selected as the study objects, and 200 cases were finally included. The patients were randomly divided into 4 groups: a placebo group, a Deanxit group (one capsule in the morning and one capsule at noon), a NeuroWell group (one capsule each time, twice a day), and a Deanxit and NeuroWell combination group, with 50 patients in each group. The demographic characteristics of the patients are shown in Table 1. Depression was diagnosed based on the patient’s history, clinical manifestations, and scale evaluations ^1^. Depending on the number and severity of symptoms, depressive episodes were classified as mild, moderate, or severe. The diagnostic criteria referred by International Classification of Diseases (ICD-10): (1) core symptoms: 1) low mood; 2) loss of interest and pleasure; 3) increased fatigue and reduced energy; (2) additional symptoms: 1) decreased attention; 2) lower self-evaluation and self-confidence; 3) self-sin and unworthiness; 4) gloomy and pessimistic future; 5) self-injury or suicidal ideation or behavior; 6) sleep disorders; 7) lower appetite. Mild depression was diagnosed in those with at least 2 core symptoms and 2 additional symptoms; moderate depression was diagnosed in those with at least 2 core symptoms and 3 additional symptoms and should have been clinically diagnosed.

The inclusion criteria were: 1) complied with the diagnostic criteria for depression, and the medical history and relevant examinations were completed; 2) aged 18-70 years; 3) 24-item Hamilton Depression Scale (HAMD-24) ≤ 35; 4) junior high school education or above; 5) provided written informed consent. The exclusion criteria were: 1) depression secondary to other mental and physical diseases; 2) severe heart, liver, or kidney functional impairment or unstable vital signs; 3) pregnant and lactating women; 4) alcoholics and drug addicts; 5) patients with serious aphasia, agnosia or who were unable to communicate; 6) serious suicidal tendencies; 7) allergy to Deanxit. The criteria for exclusion from the analysis were: 1) complied with relevant diagnostic criteria but did not cooperate with treatment; 2) developed serious complications or adverse events in response to treatment; 3) were unable to undergo venipuncture; 4) m had poor compliance due to a change of address or the addition of other treatment schemes; 5) became pregnant during treatment; 6) voluntarily withdrew from the study; 7) terminated the study due to various causes, was lost to follow-up or died.

This study protocol was approved by the Ethics Committee of Qilu Medical University (Approval No.: YXLL-KY-2021 (1)).

**Treatment Procedures**

All patients were divided into groups in a blind manner. Physicians who had been trained conducted an evaluation of the HAMD, Self-rating Depression Scale (SDS), Hamilton Anxiety Scale (HAMA), Zung Self-rating Anxiety Scale, and Pittsburgh Sleep Quality Index (PSQI) before treatment and 2, 4, 6, 8, 10, and 12 weeks after treatment to increase the consistency and objectivity of the results. The PSQI consisted of seven items with a total 21 points: higher points indicate poorer sleep quality. The HAMA score was based on 14 items. A total score < 7 indicated no anxiety, 7 - 13 indicated possible anxiety, 14 - 23 indicated anxiety, 24 - 29 indicated obvious anxiety and > 29 indicated severe anxiety. The HAMD score was based on 24 items. A score < 8 indicated no depression, 8 - 19 indicated probable depression, 20 - 35 indicated mild to moderate depression, and > 35 indicated severe depression.

**Statistical analysis**

The data were statistically processed using SPSS 25.0, and the values were expressed as the means ± standard deviation (x ± s). If the variance was homogeneous, the paired t-test was used for intra-group comparisons, and the independent sample t-test was used for intergroup comparisons. If the variance was uneven, the rank-sum test was used. If the counting data conformed to a normal distribution, the chi-squared test was used, and the nonparametric test was used for non-normally distributed data. A value of P < 0.05 was considered to indicate statistical significance.

**
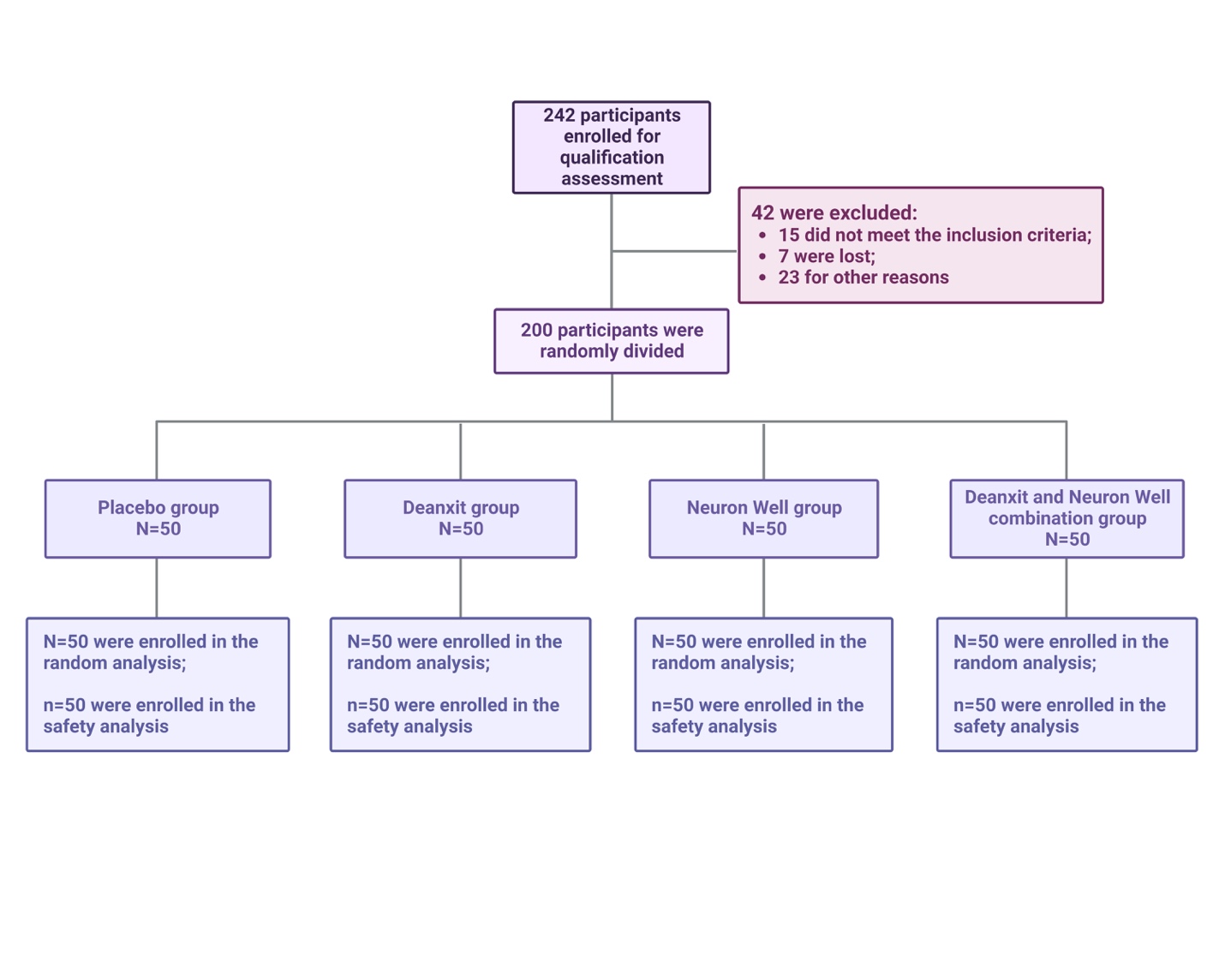
**

**Supplementary Figure 1. Flowchart demonstration of the inclusion, randomization, and follow-up of clinical participants.**


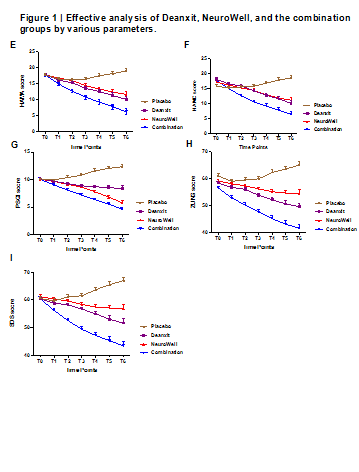

Supplement: Multimedia component 2 [file mmc2.docx]
